# Quantitative Study on Corrosion of Steel Strands Based on Self-Magnetic Flux Leakage

**DOI:** 10.3390/s18051396

**Published:** 2018-05-02

**Authors:** Runchuan Xia, Jianting Zhou, Hong Zhang, Leng Liao, Ruiqiang Zhao, Zeyu Zhang

**Affiliations:** 1 College of Civil Engineering, Chongqing Jiaotong University, Chongqing 400074, China; rcxia@mails.cqjtu.edu.cn (R.X.); hongzhang@cqjtu.edu.cn (H.Z.); 2 School of Materials Science and Engineering, Chongqing Jiaotong University, Chongqing 400074, China; lengliao@cqjtu.edu.cn (L.L.); rqzhao@cqjtu.edu.cn (R.Z.); 3 Guizhou Expressway Group Co., Ltd., Guiyang 550000, China; m15085948924@163.com

**Keywords:** corrosion, steel strand, self-magnetic flux leakage, magnetic dipole model, logistic growth model

## Abstract

This paper proposed a new computing method to quantitatively and non-destructively determine the corrosion of steel strands by analyzing the self-magnetic flux leakage (SMFL) signals from them. The magnetic dipole model and three growth models (Logistic model, Exponential model, and Linear model) were proposed to theoretically analyze the characteristic value of SMFL. Then, the experimental study on the corrosion detection by the magnetic sensor was carried out. The setup of the magnetic scanning device and signal collection method were also introduced. The results show that the Logistic Growth model is verified as the optimal model for calculating the magnetic field with good fitting effects. Combined with the experimental data analysis, the amplitudes of the calculated values (*B_xL_*(*x,z*) curves) agree with the measured values in general. This method provides significant application prospects for the evaluation of the corrosion and the residual bearing capacity of steel strand.

## 1. Introduction

The non-destructive test (NDT) [1] does not harm the measured components and structures, which is widely used in civil engineering, and other research fields. Some physical quantities or chemical parameters detected by the non-destructive instrument are used to evaluate the physical property, chemical composition or mechanical performance, providing for decision-making of structural maintenance and strengthening. 

Based on the ferromagnetic properties, methods to detect the magnetic property of steel products have emerged [2], including magnetic flux leakage (MFL) [3], magnetic acoustic emission (MAE) [4], magnetic Barkhausen noise (MBN) [5] and metal magnetic memory (MMM) [6]. During the research on MMM, the self-magnetic flux leakage (SMFL) results from different types of defects. Compared with MFL, SMFL is produced by the material itself near the defect without the tools to excite the magnetic field. When scanned along the direction parallel to the surface of steel material, the tangential component of SMFL still appears at an extreme point while the normal component still appears to have zero values [7]. The qualitative phenomenon can be obviously caught, and, therefore, how to establish the quantitative relationship between the defect and SMFL becomes the focus of the field.

The findings about SMFL of the defect have been developed for decades. The theoretical derivation and the experimental verification are two representative methods. Between the various theoretical models, the magnetic dipole model [8] is relatively vivid and practical, reflecting the magnetic field intensity of arbitrary points. The model has been deeply studied from different aspects extensively. For example, Mandalche [9] had calculated the SMFL around the single cylinder and two adjacent cylinders. Le [10] had calculated the SMFL of the hollow defect in pipes. Zhong [11,12] had studied the magnetic pole distributed with finite on the finite length and the infinite length. Shi [13] had deduced and summarized some analytic solutions of the model for a notch in different shapes (rectangle, V shape, and combination type). Wang [14] had used the model to analyze SMFL caused by the stress concentration. The experiment includes the physical experiment and the stimulation experiment (finite element analysis, FEA). Combined with experimental data, the theoretical models can be verified and are more convincing. For example, the experiment to analyze the adaptability of the model [15] had been conducted. In addition, the model had been applied to other fields, such as target location, parameter analysis and aerial survey [16]. As a quantitative model to describe the SMFL, however, the magnetic charge supposed in the model is an undetermined parameter, which is insufficient to indicate the changing law of SMFL signals.

The paper is organized as follows: in Section 2, the theoretical background of the corrosion detection is proposed, including the magnetic dipole model of SMFL and the Logistic Equation. In Section 3, the experiments of corrosion detection by the magnetic sensor are carried out and analyzed with the theoretical model, and, in Section 4, all of the results are summarized.

## 2. Theoretical Background

### 2.1. Magnetic Dipole Model-Based Corrosion Detection Method

**Figure 1:**
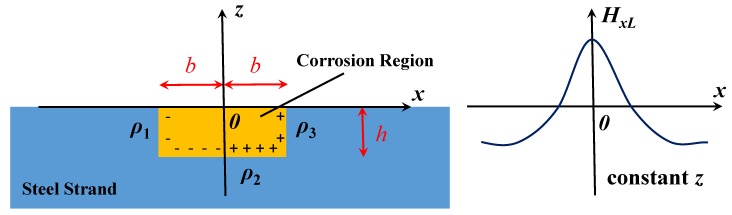
Calculated diagram and result of the magnetic dipole model.

With the conception of the dipole introduced, the magnetic dipole model emerged, which was an analogy to the electric dipole model [17]. According to the equivalent magnetic charge theory [18], the magnetic charge is the abstract physical model. It is supposed that the positive magnetic charges are gathered on the N pole, and the negative magnetic charges are gathered on the S pole. For a ferromagnet, its exterior magnetic field is considered to originate from the magnetic charge: *ρ* = −ᐁ*M* [19]. *M* is the magnetization satisfying *M =* (*μ_r_* − 1) *H_mL_*, where *μ_r_* is the relative magnetic permeability and *H_mL_* is the Weiss field, which is the effective field producing self-magnetization in the ferromagnet. In Figure 1, it is supposed that the magnetic charges are distributed on the surface of the defect. Except for the uniform distribution on the two sides (*ρ*_1_ = −*ρ*_max_ and *ρ*_3_ = *ρ*_max_), the linear distribution of the charge is designed on the bottom (*ρ*_2_ = *ρ*_max_ × *x/b*) [19,20]. Therefore, the magnetic field intensity of SMFL (*H_L_*) can be generated due to these charges. Aimed at the rectangular pit with the dimension of 2*b* × *h*, the *x*-directional component (*H_xL_*) can be quantitatively described as Equation (1) [19]. It is the general equation of the magnetic field at any point (*x*, *z*). In fact, the magnetic field is usually detected in the form of magnetic induction intensity (*B_xL_*). Correlation between *B_xL_* and *H_xL_* can be revealed by the magnetic permeability *μ* as Equation (2) [21]:

(1) $$\begin{array}{ll} H_{xL}(x,z)= & \frac{\rho_{\max}}{2\pi \mu_{0}}\left(\arctan\frac{z+h}{x+b}-\arctan\frac{z}{x+b}\right)\\ & -\frac{\rho_{\max}}{2\pi \mu_{0}}\left(\arctan\frac{z+h}{x-b}-\arctan\frac{z}{x-b}\right)\\ & +\frac{\rho_{\max}}{2\pi \mu_{0}b}\left(2b-(z+h)\left(\arctan\frac{b-x}{z+h}-\arctan\frac{-b-x}{z+h}\right)\right)\\ & +\frac{\rho_{\max}}{2\pi \mu_{0}b}\left(\frac{x}{2}\ln\frac{{\left(b-x\right)}^{2}+{\left(z+h\right)}^{2}}{{\left(b+x\right)}^{2}+{\left(z+h\right)}^{2}}\right) \end{array}$$

(2) $$B_{xL}(x,z)=\mu H_{xL}(x,z)=\mu_{0}\mu_{r}H_{xL}(x,z)$$

where *H_xL_*(*x,z*) and *B_xL_*(*x,z*) is *x*-directional component of magnetic field and induction intensity for the arbitrary point (*x*, *z*); *ρ*_max_ is the maximum magnetic charge density; *z* is the vertical coordinate, *b* is the corrosion half-width, and *h* is the corrosion depth; *μ*_0_ is the permeability of air; and *μ_r_* is the relative permeability.

Based on the hypothesis of the magnetic charge, the magnetic field distribution is well simulated through the pure mathematical deduction. Although the values of the charge are undetermined, the distribution law is coincident with the experimental phenomenon and commonly used by researchers.

### 2.2. Signal Processing for Extreme Value

To facilitate the further analysis of the experimental data, the maximum value or the extreme values, *B_xL_*_0_ and *H_xL_*_0_, are selected as the characteristic point. According to Figure 1, the curve of *H_xL_* is *z* axisymmetric, so *H_xL_*_0_ can be obtained when *x* equals 0 (Equation (3)). Meanwhile, the corresponding magnetic induction intensity *B_xL_*_0_ can be calculated (Equation (4)), and the simplified form can be expressed as Equation (5):

(3) $$\begin{array}{ll} H_{xL0}=H_{xL}(0,z) & =\frac{\rho_{\max}}{\pi \mu_{0}}\left(\arctan\frac{z+h}{b}-\arctan\frac{z}{b}\right)+\frac{\rho_{\max}}{\pi \mu_{0}}\left(1-\frac{\left(z+h\right)}{b}\arctan\frac{b}{z+h}\right)\\ & =\frac{\rho_{\max}}{\pi \mu_{0}}\left(1-\arctan\frac{z}{b}\right)+\frac{\rho_{\max}}{\pi \mu_{0}}\left(\arctan\frac{z+h}{b}-\frac{\left(z+h\right)}{b}\arctan\frac{b}{z+h}\right)\\ & =\frac{\rho_{\max}}{\pi \mu_{0}}\left(1-\arctan\frac{z}{b}+\arctan\frac{z+h}{b}-\frac{\left(z+h\right)}{b}\arctan\frac{b}{z+h}\right) \end{array}$$

(4) $$B_{xL0}=\mu_{0}\mu_{r}H_{xL0}$$

where *H_xL_*_0_ and *B_xL_*_0_ is the extreme value of *H_xL_*(*x,z*) and *B_xL_*(*x,z*),

(5) $$B_{xL0}=A\left(1-\arctan\frac{z}{b}+\arctan\xi -\xi \arctan\frac{1}{\xi }\right)$$

where *A* = *μ_r_ρ*_max_/*π*, *ξ* = (*z* + *h*)/*b*,

(6) $$h=\frac{D}{2}\left(1-\sqrt{\frac{\Delta m}{\rho Sb}}\right)$$

where *D* is the nominal diameter; ∆*m* is the accumulated mass loss; *ρ* is the density; and *S* is the nominal area of the specimen.

As can be seen from Equation (5), the physical quantity *B_xL_*_0_ is dependent on four variables (*z*, *b, h* and *A*). *z* and *b* can be directly measured. *h* can be appropriately stimulated by the actual corrosion and calculated (Equation (6)), while the unknown coefficient *A* depends on the material property *μ_r_* and the virtual quantity *ρ*_max_, which are too difficult to measure and indicate the changing law. It can be seen that *A* controls the values of *B_xL_*_0_. Based on the previous study [22,23], *B_xL_*_0_ is positively correlated to the corrosion depth *h* when *z* and *b* are constant. In this paper, three growth models are introduced to describe the correlation between *A* and *h*.

**Figure 2:**
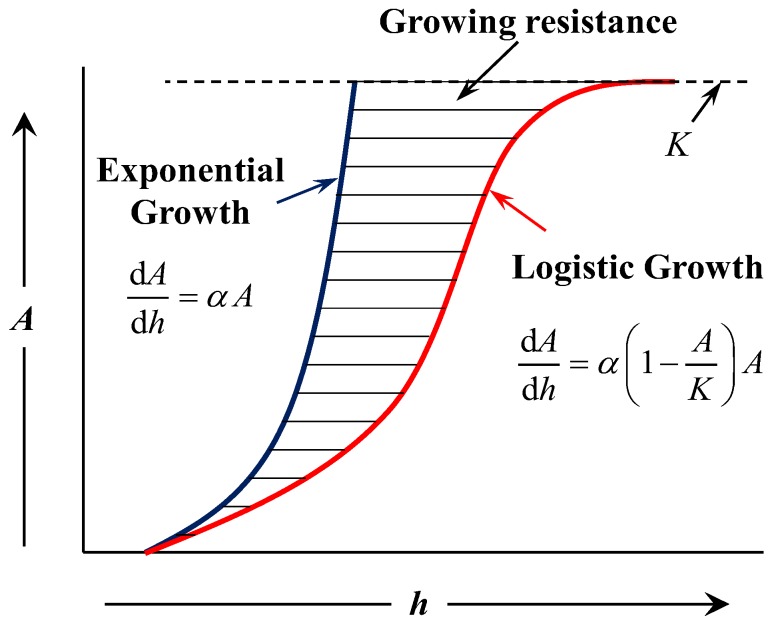
Diagram of the logistic and exponential growth model.

The original form of Logistic Equation was proposed by Malthus [24,25] in 1798. In Figure 2, Equation (7) presented the exponential growth. Afterwards, the improved model [26] was put forward by Verhulst in 1838. Taking into account the growing resistance, the growth rate would increase first and then decrease to zero. Therefore, in Figure 2, Equation (8) presented the logistic growth (S-type growth). In addition, the linear growth model was also proposed as Equation (9):

(7) $$\{\begin{array}{c} \frac{dA}{dh}=\alpha A\\ A(h_{0})=A_{0} \end{array}$$

(8) $$\{\begin{array}{c} \frac{dA}{dh}=\alpha \left(1-\frac{A}{K}\right)A\\ A(h_{0})=A_{0} \end{array}$$

(9) $$\{\begin{array}{c} \frac{dA}{dh}=\alpha \\ A(h_{0})=A_{0} \end{array}$$

where *A*_0_ is the initial value; *h*_0_ is the initial corrosion depth; *K* is the limit value of *A*; and *α* is the growth rate.

Corresponding to the three different growth models (Equations (7)–(9)), the integral equations could be respectively obtained and simplified as Equations (10)–(12):

(10) $$A_{1}=A_{0}\cdot e^{\alpha (h-h_{0})}=e^{\alpha \cdot h-\beta }$$

(11) $$A_{2}=\frac{K}{1+\frac{K-A_{0}}{A_{0}}e^{-\alpha (h-h_{0})}}=\frac{K}{1+e^{\gamma -\alpha \cdot h}}$$

(12) $$A_{3}=\alpha \cdot h+(A_{0}-\alpha \cdot h_{0})=\alpha \cdot h+\eta$$

where *β*, *γ* and *η* are three simplified parameters, *β* = *α·h*_0_ − ln*A*_0_, *γ* = *α·h*_0_ − ln (*A*_0_/(*K − A*_0_)), *η = A*_0_ − *α·h*_0_.

## 3. Experimental Study

### 3.1. Experimental Setup and Procedure

In order to verify the new equation of characteristic point *B_xL_*_0_ (Equation (5)), the experimental study on steel corrosion is performed. Five steel strand specimens of dimension 15.2–7*φ*5 are prepared, and they are labeled as 1~5#. It means that the nominal diameter is 15.2 millimeters and each strand is twisted by seven wires with a diameter of five millimeters. The mechanical parameters are also presented in Table 1. It is 82B steel and belongs to high-carbon steel. The length of specimens is 200 cm.

**Figure 3:**
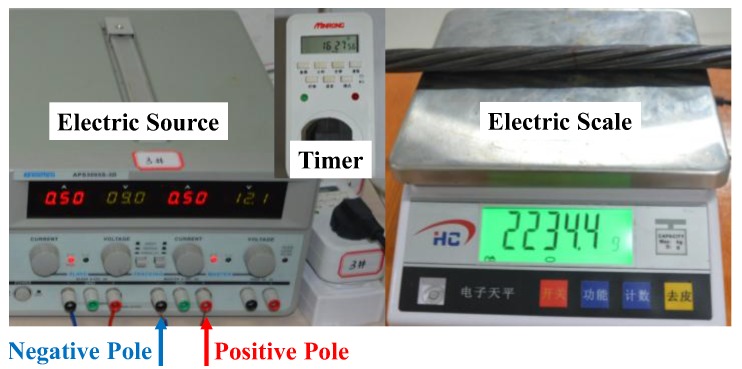
Corrosion electric source and an electric scale.

**Figure 4:**
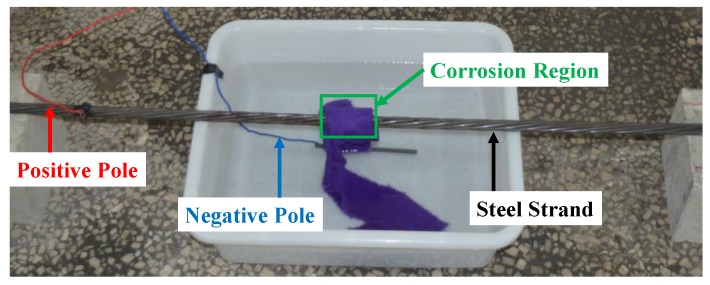
Connecting circuit based on an electrochemical corrosion method.

The electrochemical corrosion method was used to simulate the corrosion. Figure 3 shows the electric source and electric scale. Figure 4 shows the connecting circuit based on the method. The positive pole of the electric source was connected to the specimen, while the negative pole was connected to the carbon rod in the 5% sodium chloride solution. The towel wrapped around the specimen was also immersed in the solution to generate a closed loop. In that way, it would be explicit to corrode the fixed area of the specimen. To ensure the precision of the measurement, the power supply timer was adopted to set the corrosion time.

**Figure 5:**
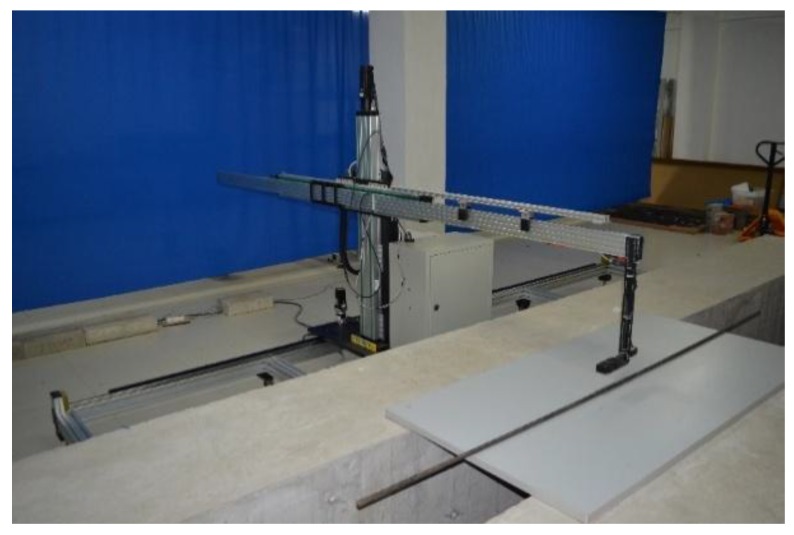
The triaxial automatic scanning device.

Figure 5 shows the triaxial automatic scanning device. The Honeywell HMR 2300 magnetometer (City, US State abbrev. if applicable, Country) [18] is installed in the device as the critical measuring sensor. The output range of the magnetometer is ±2 Gs and the resolution is 67 μGs with 10~157 sampling points per second. When the sensor moves at the speed of 300 mm/min on three orientations (the *x*-axis, the *y*-axis, and the *z*-axis), components of the magnetic induction signal (*B_x_*, *B_y_*, and *B_z_*) can be collected and saved. In general, the device has the advantage of convenient operation with a friendly control interface, which facilitates the acquisition, storage, processing and analyzing of data in the further research.

**Figure 6:**
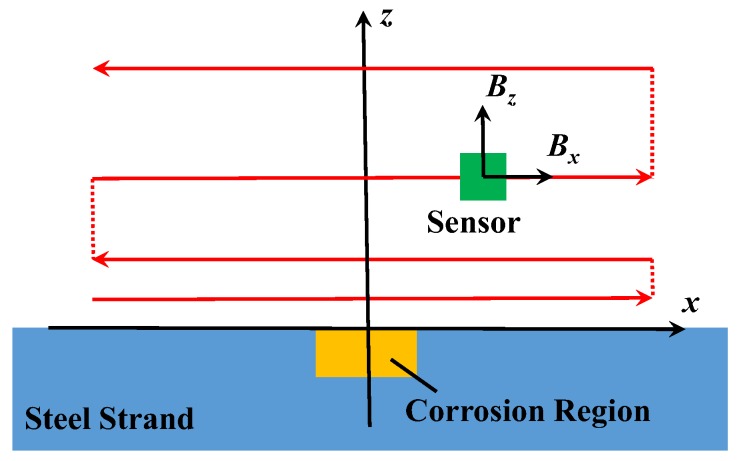
Diagram of scanning paths.

Based on the method and the device above, the experiment of the corrosion detection for the steel strand was carried out. Firstly, specimens were wrapped with towels. The width of corrosion region was 5 cm at their central positions. During the whole process of corrosion, the current of the electric source remained constant (0.5 A). In addition, 12-h corrosion was regarded as one stage. Before the specimens were scanned, the towels were taken off, and the rusts on the surface were removed together. Then, specimens in every stage were respectively weighed by the electric scale, and the mass losses could be calculated. Ultimately, the magnetic fields around were detected by the automatic scanning device. To facilitate the data process, the scanning path was parallel to the surface of the specimen in Figure 6. The solid lines indicate the scanning paths with continuous data acquisition, and the dashed lines indicate the scanning paths without data acquisition.

### 3.2. SMFL Based Corrosion Detection Results

In general, the magnetic field detected by the magnetic sensor can be mainly divided into three parts, which is presented in Equation (13). When the temperature is lower than the Curie point, the material becomes a ferromagnet, and the magnetic field related to the material is difficult to change at this time. Therefore, the magnetization of the material and the detected magnetic field are constant at room temperature. In this paper, the *x*-directional component of the total or detected magnetic induction intensity (*B_x_*) is analyzed:

(13) $$B_{x}=B_{xE}+B_{xD}+B_{xL}$$

where *B_xE_* is the environmental magnetic field; *B_xD_* is the original magnetic field generated from the material itself; and *B_xL_* is the SMFL resulted from the corrosion.

To explore the laws of *B_xL_* and combined with the horizontal scanning, *x*-*B_xL_* graphs were analyzed. According to Equation (13), *B_xL_* was only produced by the corrosion, and its value varied to different corrosion degrees. Therefore, *x*-*B_xL_* graphs were obtained with the original data subtracted by 0-h corrosion data. 

**Figure 7:**
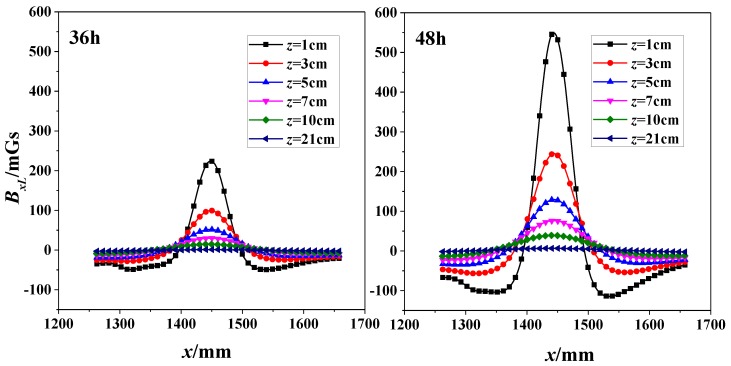
Correlation of SMFL Signal (*B_xL_*) with *x*-direction for 36-h and 48-h corrosions (1#).

Two subfigures in Figure 7 shows the correlation of SMFL signal (*B_xL_*) with *x*-direction for specimen 1# at 36-h and 48-h corrosion stages respectively. In addition, these curves differ from values of *z*. When corrosion time was 36-h, it shows that the extreme points exist in the region where the corrosion region locates. The phenomenon is also enhanced when corrosion time increases to 48 h. If the *z* value continues to increase, however, *x*-*B_xL_* curve changes gently and the extreme points dramatically decrease close to zero. 

**Figure 8:**
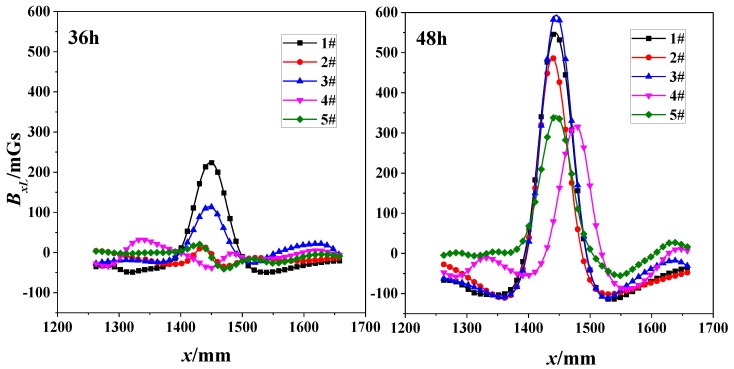
Correlation of SMFL Signal (*B_xL_*) with *x*-direction for 36-h and 48-h corrosions (z = 1 cm).

Two subfigures in Figure 8 shows the correlation of SMFL signal (*B_xL_*) with *x*-direction for the nearest scanning path (*z* = 1 cm) with 36-h and 48-h corrosions respectively. The extreme points of both 1# and 3# are evident for 36-h corrosions. However, when corrosion time increases to 48 h, the phenomenon is enhanced and suitable for all specimens. Some diversities still exist with the same changing law of curves. This may be caused by the manufacture of specimens or the material itself, such as the internal twisting force, the different initial defect or the initial magnetization degree.

In a word, it can be preliminarily determined that the SMFL is positively correlated with the corrosion degree, but negatively with *z*-value.

### 3.3. Quantitative SMFL Signal Analysis Using Magnetic Dipole Model

The following research content is to quantitatively analyze the extreme values of the SMFL signal and verify the theoretical equation. Firstly, the actual corrosions at different corrosion stages were weighed and listed in Table 2. According to Equation (6), the corresponding corrosion depth *h* could also be obtained and the calculated values were shown in the brackets after the actual corrosions in Table 2. Then, based on the common feature of Figure 7, the extreme points of *x*-*B_xL_*_0_ curves were extracted, which could be seen in Table 3. In experiments, *z* is called the lift-off height (the distance between the magnetic sensor and surface of the specimen).

**Figure 9:**
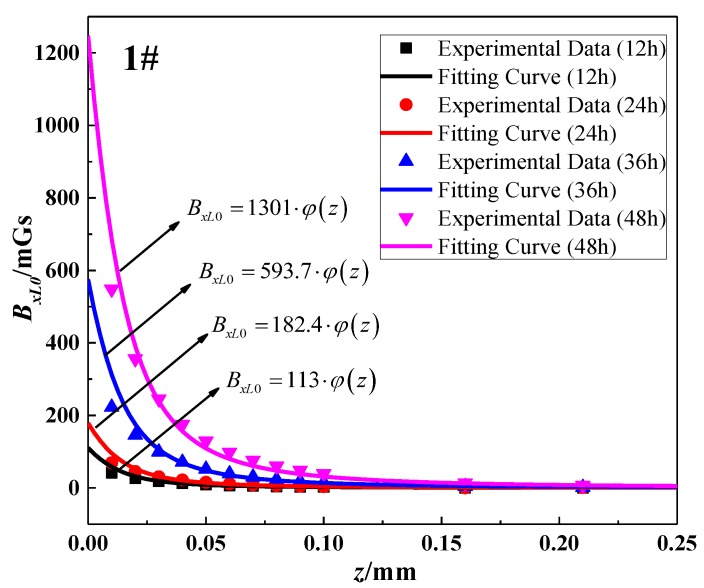
Fitting effect of *z-B_xL0_* curve for specimen 1#.

Table 3 presented the extreme values of SMFL corresponding to each corrosion time. Aimed at the data in one column, the values of *b* and *h* were constant. Therefore, *B_xL_*_0_ was only dependent on *A* and *z*, and Equation (5) could be simplified as Equation (14). By using the data fitting method, the value of *A* to different corrosion degrees could be obtained that the theoretical model would coincide with experimental data. Partial fitting curves and their corresponding experimental data (12 h, 24 h, 36 h, and 48 h) were shown in Figure 9. To sum up, all results of *A* were listed in Table 4. In addition, the statistics *R*^2^ is the coefficient of determination to measure the goodness of fitting. *R*^2^ approaching 1 means the better fitting effect. As is shown in Table 4, the values of *R*^2^ are greater than 0.97, which indicates that the function form (Equation (14)) can be in good agreement with the experimental data at each corrosion stage:

(14) $$B_{xL0}=A\cdot \varphi \left(z\right)$$

**Figure 10:**
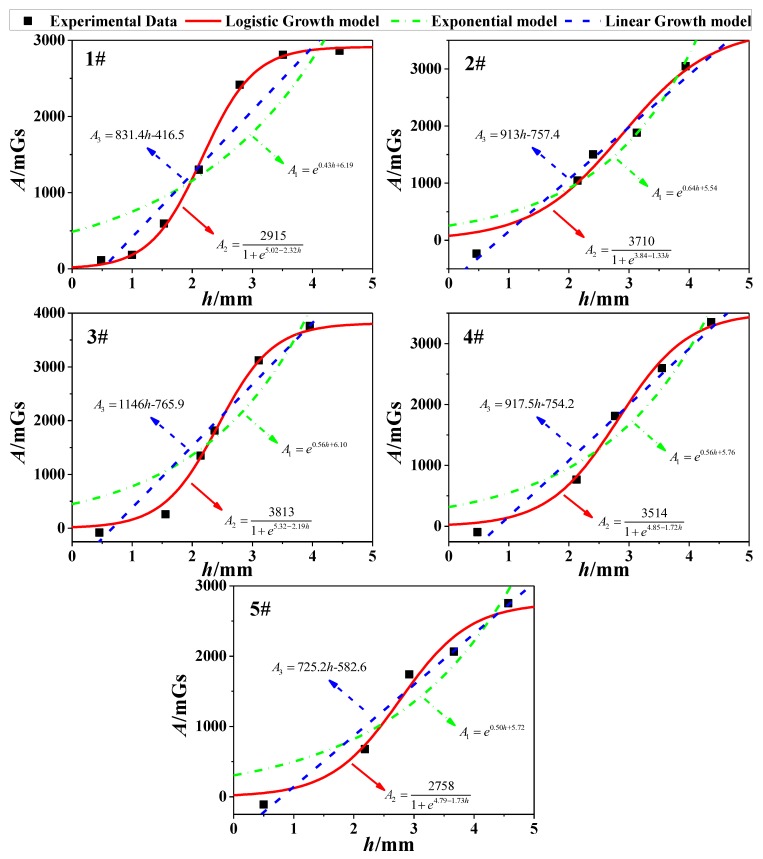
Fitting effect of *h-A* curve for specimens 1~5#.

Next, as for all corrosion stages in Table 4, the three growth models (Equations (10)–(12)) were used to show the correlation between *h* and *A*. Via the fitting analysis, the fitting results of three models for 1~5# specimens could be obtained, which were shown in five subfigures of Figure 10. Meanwhile, *R*^2^ was also used to determine the goodness of fitting. As shown in Table 5, the logistic growth model is in better agreement with the experimental data compared with other models. The phenomenon could be explained from the point of the magnetic signal strength. In the initial stage of corrosion, SMFL will produce at the increasing speed. However, when the corrosion increases constantly, the distance between the corrosion surface and the magnetic sensor will also increase, which results in the signal decay. Thus, the value of SMFL will increase at the decreasing speed. In a word, Equation (11) was verified as the optimal model for calculating the magnetic field.

As for Figure 10, the differences of *A* are diverse in the same order of the magnitude. The phenomenon may result from the magnetization degree of specimens, the diversity of the material or the internal twisting force of the steel strand.

**Figure 11:**
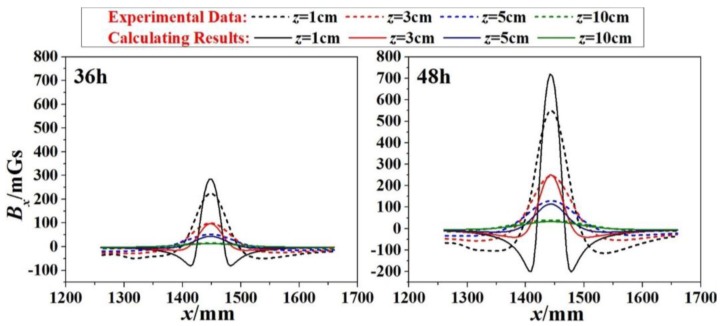
Comparison between the experimental data and the calculated results for 36-h and 48-h corrosions (1#).

To verify the effect of the proposed calculation model, specimen 1# was taken as an example. As was shown in Figure 10, the calculation of *A* for specimen 1# was given as Equation (15). Therefore, combined with Equations (1), (2) and (15), the calculated magnetic induction intensity *B_xL_*(*x,z*) could be obtained. The parameters of *B_xL_*(*x,z*) were clarified (*b* = 25 mm, *h* = 1.53 mm for 36-h corrosions, *h* = 2.11 mm for 48-h corrosions, *z* = 1 cm, 3 cm, 5 cm and 10 cm). Therefore, the results for 36-h and 48-h corrosions were shown in Figure 11, where the dashed lines present the experimental data and the solid lines present the calculating results. When the lift-off height *z* is small, the signal is more sensitive to the *z*, and the difference between them appears to be little bigger. However, in general, the amplitude of the calculated values agrees with the measured values:

(15) $$\frac{\mu_{r}\rho_{\max}}{\pi }=A_{2}=\frac{2915}{1+e^{5.02-2.32h}}$$

## 4. Conclusions

In the magnetic detection of the corrosion, the Self-Magnetic Flux Leakage (SMFL) is the key to the analysis. In this study, the distribution of SMFL (*B_xL_*(*x,z*)) was calculated based on the original magnetic dipole model. Intuitively, the characteristic point of the signal (extreme point *B_xL_*_0_) is used to uncover the information of corrosions. In addition, three growth models (logistic model, exponential model, and linear model) were proposed to explore the changing law of the unknown coefficient *A* in the magnetic dipole model. Then, the experimental study on corrosion detection of the steel strand was carried out and analyzed with the theoretical models. Finally, the following conclusions can be drawn:

(1) Combined with the fitting analysis of the experimental data, the Logistic Growth model is in better agreement with the correlation between *h* and *A*, and it is verified as the optimal model for calculating the magnetic field (Figure 10).
(2) With the logistic model brought to the original magnetic dipole model, the amplitudes of the calculated values (*B_xL_*(*x,z*) curves) coincide with the measured values in general (Figure 11).
(3) The differences of *A* for specimens are diverse in the same order of the magnitude. This may be caused by the manufacture of specimens or the material itself, such as the internal twisting force, the different initial defect or the initial magnetization degree.

The paper provides prospects for the quantitative research in the non-destructive magnetic detection technology. In the further in-depth study, adopting more specimens in the experiment will help obtain the stronger regularity. In that case, the value of *A* can be optimized through the data integration and some processing methods as the normalization. This will meet the requirements of estimating the magnetic field without the experiment in a certain range of errors.

## Figures and Tables

**Table 1:** Basic parameters of steel strand specimens.

Nominal Diameter *D*/mm	Tensile Strength/MPa	Limit Load *F_b_*/kN	Yield Load *F_y_*/kN
15.2	1860	259	220

**Table 2:** Accumulated mass losses and calculated corrosion depth *h* of specimens. (unit: g and mm).

	Label	1#	2#	3#	4#	5#
Corrosion Time						
12 h		6.8 (0.48)	6.5 (0.46)	6.4 (0.46)	6.7 (0.48)	6.7 (0.48)
24 h		13.5 (1.00)	13.3 (0.98)	13.2 (0.97)	13.5 (1.00)	13.8 (1.02)
36 h		19.9 (1.53)	20.3 (1.56)	20.2 (1.55)	20.3 (1.56)	20.4 (1.57)
48 h		26.3 (2.11)	26.7 (2.15)	26.6 (2.14)	26.5 (2.13)	27.1 (2.18)
60 h		33.0 (2.79)	29.3 (2.40)	29.0 (2.37)	32.8 (2.77)	34.2 (2.92)
72 h		39.1 (3.54)	36.0 (3.13)	35.8 (3.11)	39.4 (3.55)	40.3 (3.67)
84 h		45.6 (4.45)	42.3 (3.94)	42.4 (3.96)	45.1 (4.37)	46.3 (4.57)

**Table 3:** Extreme values *B_xL_*_0_ for specimen 1# (unit: mGs).

	Corrosion Time	12 h	24 h	36 h	48 h	60 h	72 h	84 h
z/m								
0.01		41.4	69.4	222.5	548.2	1031.8	1180.2	1204.1
0.02		26.6	45.3	145.6	356.0	671.1	793.0	827.1
0.03		18.0	30.5	99.1	245.0	467.6	557.3	586.5
0.04		12.6	21.6	70.8	174.8	338.5	410.4	436.9
0.05		9.1	15.4	51.7	129.3	252.9	310.2	332.5
0.06		6.7	11.2	39.0	98.0	193.4	240.7	259.7
0.07		5.1	8.3	29.9	76.2	151.5	190.2	206.0
0.08		3.8	6.1	23.1	60.0	120.5	152.9	166.4
0.09		2.9	4.6	18.4	48.3	97.6	124.9	136.1
0.10		2.1	3.2	14.5	39.0	79.8	103.0	112.8
0.16		0.2	0.1	4.0	13.7	29.4	39.6	43.5
0.21		0.4	0.8	1.3	6.6	15.3	21.3	22.9

**Table 4:** Fitting results of *z-B_xL0_* curve for specimen 1#.

Corrosion Time	12 h	24 h	36 h	48 h	60 h	72 h	84 h
*h*/mm	0.48	1.00	1.53	2.11	2.79	3.51	4.45
*A*	111.3	182.4	593.7	1301	2416	2810	2864
*R* ^2^	0.9949	0.9952	0.9942	0.9856	0.9827	0.9771	0.9745

**Table 5:** Results of *R*^2^.

	1#	2#	3#	4#	5#
Logistic Growth model	0.9979	0.9527	0.9979	0.9911	0.9766
Exponential Growth model	0.7894	0.9221	0.9000	0.9215	0.8912
Linear Growth model	0.9245	0.9768	0.9714	0.9666	0.9696
